# Doctors and Education Committees

**Published:** 1903-01-10

**Authors:** 


### Doctors and Education Committees.

The various county, county borough, and urban district councils, which, under the new Act, have become the local education authorities for their respective areas of government, are charged, as their first duty in that capacity, with the work of preparing, and of submitting to the Central Board, schemes for the constitution of the education committees through which most of their powers will be exercised under the Act. In view of the facts, now generally recognised even by comparatively ignorant people, that health and education are more or less correlative, and that conditions injurious to the former must inevitably impede the progress of the latter, it has become important that the sanitary condition of schools should receive the earnest attention of all who are concerned in conducting them; and it is manifest that the only way in which this attention can be properly directed and usefully bestowed will be by the assistance of persons qualified by special knowledge to deal with the problems which from time to time will inevitably offer themselves for solution. In these circumstances, it is not too much to hope that every council will be wise enough to seek to place at least one medical practitioner of the locality upon its committee, and also that, in every locality, at least one practitioner will be found with sufficient public spirit to undertake the duty, and to devote the necessary time and thought to its fulfilment. Parliament has left the councils a tolerably free hand in the constitution of their committees, but has safeguarded the exercise of the power thus bestowed by providing that a control shall be exerted by the Board of Education, whose approval will be necessary for every scheme, and who are required to make each scheme public, with a view to its being subjected to local criticism before its acceptance, rejection, or modification, is finally decided upon. The object of publication is to call the attention of residents in each locality to the persons nominated as members of the committee by the council, with a view to the sending in of suggestions; so that, in the event of a council having forgotten or failed to include anyone whose presence would manifestly be useful or desirable, such an omission may be mentioned to the central board by any ratepayer of the district. In any case, therefore, in which a medical man is willing to act upon the local committee, but is not included in the list of names submitted by the council, it will be open to anyone interested to bring his name, and his willingness to serve, under the notice of the Education Board.

In some strange way the medical profession has been led to endeavour to hold a middle course on many important questions in order to avoid the possibility of any conflict between their feelings as citizens and the due discharge of their duties as practitioners. The chief objections to such an arrangement must rest upon its obvious insincerity, in view of the demand it makes that members of the profession should sacrifice genuine convictions for the sake of appearing to preserve a neutrality which everyone concerned would know to be unreal. Is it not time that the pretence should be abandoned, and that the doctor, like every other good citizen, should be prepared not only to take his share of public duty, but to claim as a matter of right that this share should be allotted to him. We suggested last week that the doctor might usefully take the initiative in calling upon the wage-earning classes of this country to aim at independence during sickness; and we would now urge that he should insist upon his right to exercise a due share of control in questions connected with the education of children, on many of which his special knowledge would enable him to speak with a degree of authority which no other class of persons can possess. Education, after all, is only a matter of applied physiology; and the applications of physiology can only be made by physiologists. Sir James Crichton Browne, Dr. Dukes, and many other authorities, have shown how close is the connection between bodily health and intellectual capabilities; how essential to the scholar is the provision of proper food and the avoidance of undue fatigue; how important is physical culture in all its forms, and how far-reaching may be the effects of neglect of the laws which Nature has plainly written for the guidance of all who will observe them. We repeat that the place of the doctor in education is one which cannot be filled by the members of any other calling, and that, although the doctor has confessedly much to learn with regard to it, he is the only member of the community who possesses the groundwork upon which that further learning can be based. We trust that he will claim his right to an important place on the education committees of the future, and that by doing so he may pave the way to a more general recognition of his rights as a citizen, and of the value of his special knowledge, not in the sick-room only, but in the general guidance of a large proportion of public affairs.

